# Wound healing properties of a new formulated flavonoid-rich fraction from *Dodonaea viscosa* Jacq. leaves extract

**DOI:** 10.3389/fphar.2023.1096905

**Published:** 2023-02-02

**Authors:** Shanthi Subramanian, Chamundeeswari Duraipandian, Abdulrhman Alsayari, Gobinath Ramachawolran, Ling Shing Wong, Mahendran Sekar, Siew Hua Gan, Vetriselvan Subramaniyan, S Seethalakshmi, Srikanth Jeyabalan, Sivaraman Dhanasekaran, Suresh V. Chinni, Nur Najihah Izzati Mat Rani, Shadma Wahab

**Affiliations:** ^1^ Department of Pharmacognosy, Sri Ramachandra Faculty of Pharmacy, Sri Ramachandra Institute of Higher Education and Research (DU), Chennai, Tamilnadu, India; ^2^ Department of Pharmacognosy, College of Pharmacy, King Khalid University, Abha, Saudi Arabia; ^3^ Complementary and Alternative Medicine Unit, King Khalid University, Abha, Saudi Arabia; ^4^ Department of Foundation, RCSI and UCD Malaysia Campus, Georgetown, Penang, Malaysia; ^5^ Faculty of Health and Life Sciences, INTI International University, Nilai, Malaysia; ^6^ Department of Pharmaceutical Chemistry, Faculty of Pharmacy and Health Sciences, Royal College of Medicine Perak, Universiti Kuala Lumpur, Ipoh, Perak, Malaysia; ^7^ School of Pharmacy, Monash University Malaysia, Subang Jaya, Malaysia; ^8^ Faculty of Medicine, Bioscience and Nursing, MAHSA University, Jenjarom, Malaysia; ^9^ Department of Pharmacology, ESIC Medical College and PGIMSR, Chennai, Tamilnadu, India; ^10^ Department of Pharmacology, Sri Ramachandra Faculty of Pharmacy, Sri Ramachandra Institute of Higher Education and Research (DU), Chennai, Tamilnadu, India; ^11^ School of Technology, Pandit Deendayal Energy University, Gandhinagar, Gujarat, India; ^12^ Department of Biochemistry, Faculty of Medicine, Bioscience, and Nursing, MAHSA University, Kuala Lumpur, Malaysia; ^13^ Department of Periodontics, Saveetha Dental College and Hospitals, Saveetha Institute of Medical and Technical Sciences, Chennai, India; ^14^ Faculty of Pharmacy and Health Sciences, Royal College of Medicine Perak, Universiti Kuala Lumpur, Ipoh, Perak, Malaysia

**Keywords:** *Dodonaea viscosa*, flavonoids, wound healing, skin infections, topical ointment

## Abstract

**Background:** *Dodonaea viscosa* Jacq. (*D. viscosa*) belongs to the family of Sapindaceae, commonly known as “*Sinatha,*” and is used as a traditional medicine for treating wounds due to its high flavonoids content. However, to date there is no experimental evidence on its flavonoid-rich fraction of *D. viscosa* formulation as an agent for healing wounds.

**Objective:** The present study aimed to evaluate the wound healing effect of ethyl acetate fraction of *D. viscosa* leaves on dermal wounds.

**Methods:** The ethyl acetate fraction was produced from a water-ethanol extract of *D. viscosa* leaves and was quantitatively evaluated using the HPLC technique. The *in-vivo* wound healing ability of the ethyl acetate fraction of *D. viscosa* ointment (DVFO, 2.5%w/w and 5%w/w) was investigated in *Sprague-Dawley* rats utilizing an incision and excision paradigm with povidone-iodine ointment (5% w/w) as a control. The percentage of wound closure, hydroxyproline and hexosamine concentrations, tensile strength and epithelialization duration were measured. Subsequently, histopathology analysis of skin samples as well as western blots were performed for collagen type 3 (COL3A1), basic fibroblast growth factor (bFGF) and vascular endothelial growth factor (VEGF).

**Results:** The ethyl acetate fraction of *D. viscosa* revealed flavonoids with high concentrations of quercetin (6.46% w/w) and kaempferol (0.132% w/w). Compared to the control group, the DVFO (2.5% and 5.0% w/w) significantly accelerated wound healing in both models, as demonstrated by quicker wound contraction, epithelialization, elevated hydroxyproline levels and increased tensile strength. Histopathological investigations also revealed that DVFO treatment improved wound healing by re-epithelialization, collagen formation and vascularization of damaged skin samples. Western blot analysis further demonstrated an up-regulation of COL3A, vascular endothelial growth factor and bFGF protein in wound granulation tissue of the DVFO-treated group (*p* < 0.01).

**Conclusion:** It is concluded that flavonoid-rich *D. viscosa* ethyl acetate fraction promotes wound healing by up-regulating the expressions of COL3A, VEGF and bFGF protein in wound granulation tissue. However, extensive clinical and pre-clinical research on the flavonoid-rich fraction of *D. viscosa* is needed to determine its significant impact in the healing of human wounds.

## Graphical Abstract

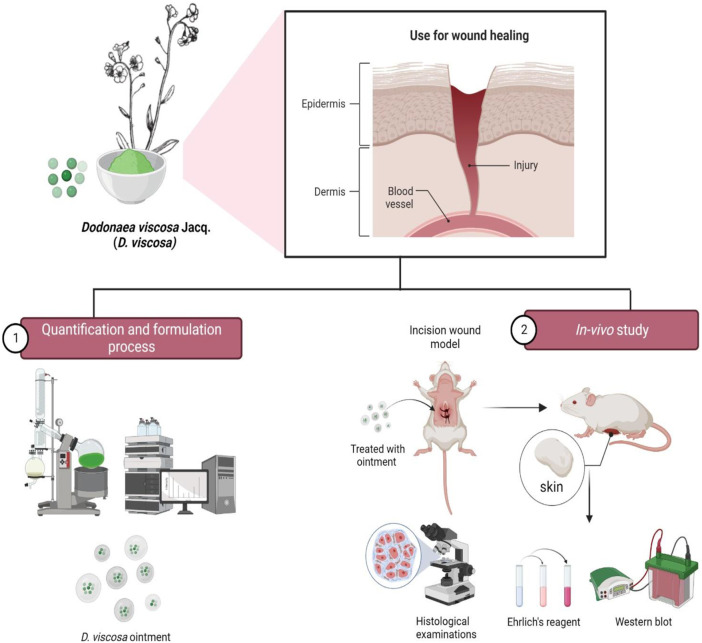

## 1 Introduction

A wound is a breakdown in the tissue’s cellular and anatomic continuity that results in the loss of epithelial continuity regardless of the loss of the underlying connective tissue. It comprises of tissue/organ damage caused by disease, pressure, friction/shear force, heat/cold, surgery, chemicals and a blow or a cut (Lawrence, 1998; Rodrigues et al., 2019; Mohd Zaid et al., 2022). Wound healing results from a complicated interplay between epidermal and dermal cells, the extracellular matrix (ECM), regulated angiogenesis and plasma-derived proteins, all of which are influenced by various cytokines and growth factors. The dynamic process includes four overlapping, continuous phases: hemostasis, inflammation, proliferation, and remodeling (Richardson, 2004; Gonzalez et al., 2016; Nourian Dehkordi et al., 2019). The first phase of hemostasis occurs shortly after injury, with vascular constriction and the creation of a fibrin clot. Alpha granules released from platelets secretes the growth factors such as platelet derived growth factor (PDGF), transforming growth factor-β (TGF-β), epidermal growth factor (EGF) and insulin-like growth factor (IGF) which act as promoters in wound healing cascade by activating and attracting neutrophils and later macrophages, endothelial cells, and fibroblasts. The inflammatory phase is distinguished by neutrophil and monocyte infiltration as well as differentiation to macrophage and lymphocyte penetration. In addition, these macrophages commence the growth of granulation tissue and discharge a variety of proinflammatory cytokines (Interleukin, IL-1 and IL-6) and growth factors [Fibroblast Growth Factor (FGF), EGF, TGF-β, and PDGF]. Fibroblasts, endothelial cells, and keratinocytes multiply during the proliferative phase, as does the deposition of fibronexus and collagen type III (Henry and Garner, 2003). This phase is characterized by fibroblast migration, deposition of newly synthesized extracellular matrix (ECM), angiogenesis and abundant formation of granulation tissue which are regulated by FGF and vascular endothelial growth factor (VEGF). Finally, during the modeling phase, collagen type III fibers are massively replaced by collagen type I fibers, more resistant to enormous pressures.

During the tissue-modeling phase, there is an increase in crosslinking among the collagen fiber monomers in the direction of the skin stress lines. Smoking, obesity, sex hormones, stress, age, nutrition, medicine, and infection may all adversely influence this process and cause delayed wound healing (Guo and DiPietro, 2010). Consequently, the therapeutic identification of compounds with healing action may differ depending on the stage of the healing process. Anti-inflammatories (steroidal and non-steroidal) and chemotherapeutics (antiseptics and antibiotics) are two often-used treatments that have a substantial influence on wound healing (Guo and DiPietro, 2010). However, they are expensive and risky, sometimes ineffective; adverse effects are often observed. As a result, herbal therapy emerges as an alternative technique for wound care (Budovsky et al., 2015). The use of various herbs and traditional medicine should also be economical in the face of escalating healthcare costs. Hence, research is essential to understand proper utilization of this nature throve to increase the number of armamentariums in advanced wound care techniques.

**FIGURE 1:**
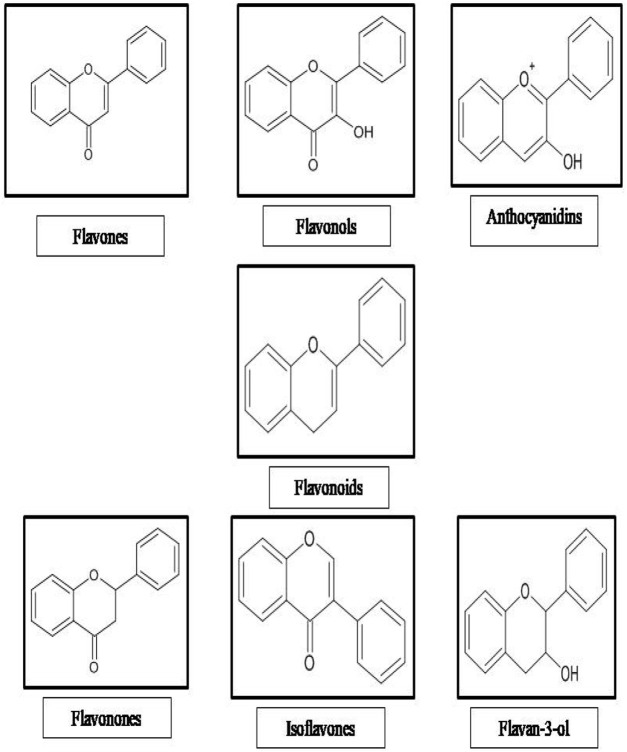
Classification of flavonoids.

Flavonoids are secondary metabolites of polyphenolic in nature. Flavonoids have fifteen carbon skeletons consisting of two benzene rings connected by a heterocyclic pyran ring. Flavonoids are categorized as flavanones, flavones, isoflavones, flavonols, flavan-3-ol, and anthocyanin (Figure 1). These compounds are coupled with multifaceted health benefits ensue from their bioactive properties, such as anti-inflammatory, anticancer, anti-aging, cardio-protective, neuroprotective, immunomodulatory, antidiabetic, antibacterial, antiparasitic, antiviral and wound healing properties in humans (Fraga et al., 2019; Jucá et al., 2020; Saini et al., 2017). Several studies have demonstrated the significance of flavonoids as wound healing agents attributed to their antioxidant, antimicrobial and anti-inflammatory properties. Furthermore, flavonoids influences the inflammatory process, angiogenesis, re-epithelialization and oxidative stress and also stimulates the macrophages, fibroblasts and endothelial cells through the expression of TGF-β1, VEGF, Ang, Tie, Smad 2 and 3, and IL-10 (Carvalho et al., 2021). Several lines of evidence indicating the wound healing potential of flavonoid rich fraction of medicinal plants *viz. Tephrosia purpurea* (L.) Pers., *Martynia annua* L., *Ononidis radix* L., and *Eugenia pruniformis* Cambess (Lodhi et al., 2013; Lodhi and Singhai, 2013; de Albuquerque et al., 2016; Ergene Öz et al., 2018)*.* Thus, medicinal plants rich in flavonoids are suitable candidates for developing newer wound healing agents from natural sources.

**FIGURE 2:**
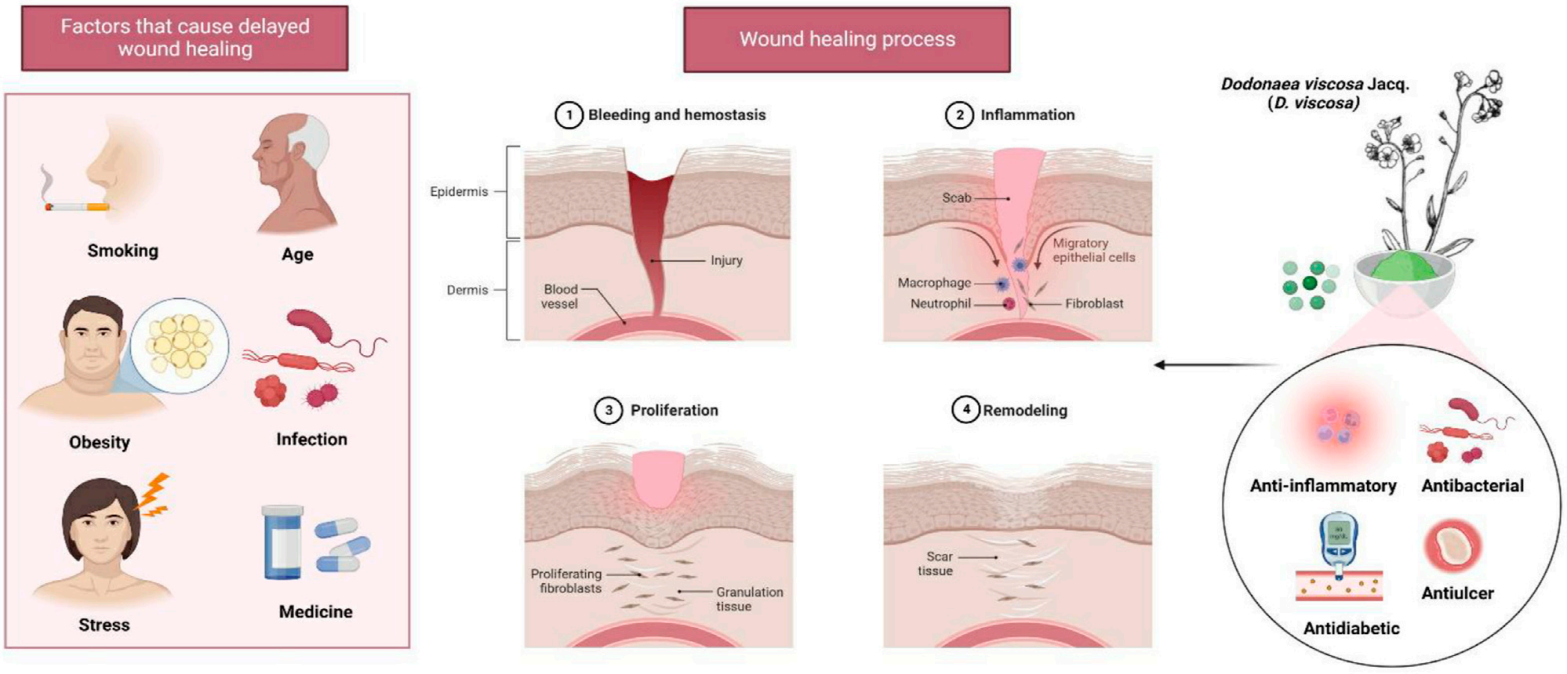
Wound healing properties of *D. viscosa* leaves. Stress and obesity are two of the many factors that might contribute to delayed wound healing. The plant’s phytoconstituents, which have a variety of properties including anti-inflammatory, antibacterial, antidiabetic, and antiulcer properties, may be useful for the treatment of infected wounds.

The presence of several flavonoids was revealed in the leaves and aerial parts of *Dodonaea viscosa* Jacq (*D. viscosa*) (Family: Sapindaceae). The plant is believed to possess anti-inflammatory, anti-bacterial and antipyretic effects and is traditionally used to treat gout, rheumatism, snakebite, wounds, swellings, and burns (Kirtikar and Basu, 1995; Mashelkar, 2008). To date, limited investigations on the pharmacological characteristics of *D. viscosa* leaves exists with scattered reports of anti-bacterial (Getie et al., 2003), antiulcer (Aruna and Asha, 2008), antidiabetic (Veerapur et al., 2010), anti-inflammatory and analgesic activities (Getie et al., 2003; Khalil et al., 2006) (Figure 2). Some important flavonoid constituents such as quercetin, kaempferol, 3,3′,4′,5,7-Pentahydroxy flavane, 5-Hydroxy-7,3′4′-trimethoxy-6-acetoxy-3-prenylflavone, 3,4′-Dimethoxy-5,7-dihydroxyflavone, 3,5,7-Trihydroxy-4′-methoxyflavone, Pinocembrin and Penduletin have been isolated (Table 1) (Getie et al., 2000; Teffo et al., 2010; Asila and Hossain, 2018; Al-Aamri and Hossain, 2016; Muhammad et al., 2015; Mostafa et al., 2014). Beshah et al. reported that many species of *Dodonaea* genus were found to possess flavonoids and terpenoids (Beshah et al., 2020). According to the findings of Sebelemetja et al. (2020) using *D. viscosa* extract, created polymeric nanoparticle which was evaluated against the oral cavity acid-producing *Streptococcus mutans* (Sebelemetja et al., 2020). Joshi et al. proved the preliminary wound healing studies with ethanol extract of *D. viscosa* leaves (Joshi et al., 2003). Nayeem et al. evaluated the wound healing potential of *D. viscosa* formulation prepared with methanol and chloroform extract of leaves in experimental animals (Nayeem et al., 2021). Literature studies indicate that *D. viscosa* leaves possess significant wound healing potential. However, the wound healing studies are lacking in the fractions of leaves and the correlation to its phytoconstituents. Our preliminary studies proved the wound healing potential of flavonoid rich fraction of *D. viscosa* by *in-vitro* cell proliferation assay on HACAT cell line (Shanthi et al., 2015). Although the plant is traditionally claimed to have fast wound-healing effects besides its rich flavonoid content, there has been no scientific data to confirm this claim on flavonoid rich fraction of *D. viscosa*. Hence, the present study aimed to investigate the mechanism of action and the wound healing properties of the flavonoid-rich ethyl acetate fraction of *D. viscosa* leaves in the forms of new topical formulations by using animal models.

**TABLE 1:** Reported flavonoids from *D. viscosa*.

Flavonoid	Chemical structure	References
Quercetin	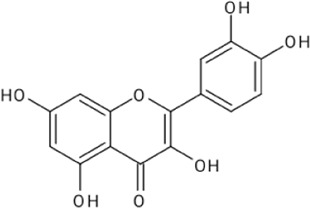	Getie *et al.* (2000)
Kaempferol	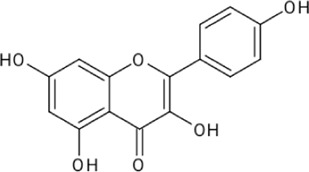	Teffo et al. (2010)
3,3′,4′,5,7-Pentahydroxy flavane	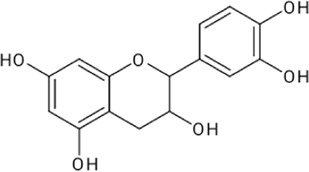	Asila and Hossain, (2018)
5-Hydroxy-7,3′4′-trimethoxy-6-acetoxy-3-prenylflavone	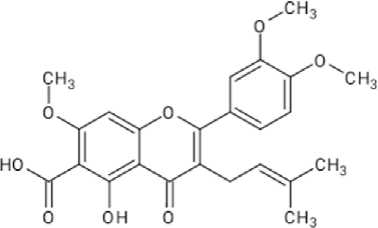	Khaloud and Hossain (2016)
3,4′-Dimethoxy-5,7-dihydroxyflavone	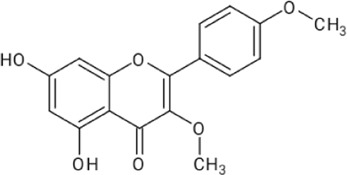	Muhammad et al. (2015)
3,5,7-Trihydroxy-4′-methoxyflavone	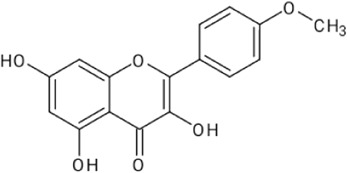	Teffo et al. (2010)
Pinocembrin (5,7dihydroxyflavone)	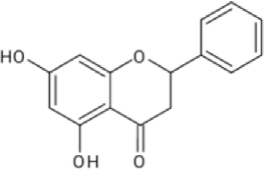	Mostafa *et al.* (2014)
Penduletin (5,4′-dihydroxy-3,6,7-trimethoxyflavone)	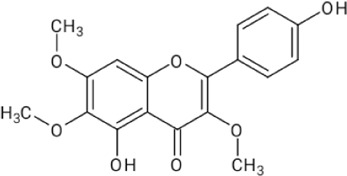	Mostafa *et al.* (2014)

## 2 Materials and methods

### 2.1 Collection, authentication and extraction of *D.vicosa*

Fresh, healthy leaves of *D. vicosa* were harvested from the Madurai district, Tamil Nadu, India. The plant was authenticated, and the herbarium was deposited at the Plant anatomical research center, Chennai (PARC/14/2169). The plant material was shade-dried and then ground into a coarse powder. The coarse powder was extracted with ethanol (70%) at room temperature for 24, 48 and 72 h. The mixed extracts were concentrated using a rotavapor to yield the final solvent-free crude extract. To get a flavonoid-rich fraction, the extract was suspended in water and separated with ethyl acetate (Seyfi et al., 2010).

### 2.2 Quantification of flavonoid by high-performance liquid chromatography (HPLC) analysis

Ethyl acetate fraction (10 mg) was refluxed for 135 min with 78 ml of alcohol, water, and hydrochloric acid cocktail (50:42:8). Subsequently, 20 ml of 70% methanol was added, followed by a sonication step for 30 min. The extract was filtered and diluted with ethanol to 100 ml. An aliquot (5 ml) was filtered through a C-18 silica column followed by methanol elution (4 ml). The elution volume was limited to 10 ml before being analyzed using a HPLC (Oliveira et al., 2001).

HPLC analysis was conducted at room temperature in a Shimadzu apparatus outfitted with an SPD-M10A diode array detector and a reverse-phase column (Linchosorb RP-18, 25 cm × 5 mm). Elution was conducted using a solution of methanol, water, and phosphoric acid (50.0:49.6:0.4). The flow rate was set at 1 ml/min, with detection set at 270 nm. Quercetin and kaempferol were utilized as controls by diluting the standards in methanol (1 mg/ml). For analysis, 10 μL of sample and standards were injected each time. The peak regions of the HPLC chromatogram from the triple selection were used to compute the flavonoids concentration.

### 2.3 Preparation of topical formulation and ethical approval

Prior to the animal study, the ethyl acetate fraction of *D. viscosa* was formulated as a *D. viscosa* ointment (DVFO) (2.5% and 5% w/w) using a simple ointment base. The experimental protocols were approved by the Institutional Animal Ethical Committee, Sri Ramachandra Institute of Higher Education and Research (Approval no: IAEC/XXXV/SRU/309/13). The approval is constituted under the Committee for the Purpose of Control and Supervision of Experimental Animals (CPCSEA-837/ac/2004) guidelines.

### 2.4 Acute dermal toxicity study

An acute dermal toxicity study was performed according to the Organization for Economic co-operation and Development (OECD) test guideline 402 (OECD, 1987). Briefly, healthy adult *Sprague Dawley* male and female rats (210–280 g) were housed individually in a polypropylene cage with proper ventilation and were randomized into a group of 10 (five animals/each gender) each. The animals were maintained at 22°C ± 3°C with a relative humidity of 50%–80% and an artificial photoperiod (12 h light/dark).

The fur was removed from the animal’s dorsal region. Then, 5% w/w DVFO formulation was evenly applied to a small area [Not less than 10% of the body surface area) of the closely clipped skin of the animals. Mortality and aberrant clinical indicators were monitored daily for 14 days until the study was completed. The body weight was measured before medication administration and then weekly until the investigation was completed. At the end of the experiment, any significant pathological variations were identified.

### 2.5 Evaluation of *in-vivo* wound healing activity

#### 2.5.1 Excision wound model

The animals were randomly divided into four groups (*n* = 12). Group I served as a control (ointment base alone), while Group II was treated with a standard (povidone iodine ointment 5% w/w). Groups III and IV were treated with DVFO 2.5% w/w and 5.0% w/w, respectively.

Before inflicting the experimental wounds, the animals were anesthetized by intraperitoneal (i.p.) injection of sodium phenobarbitone (40 mg/kg). The dorsal fur was shaved and a circular stainless-steel stencil with methylene blue was used to outline the projected wound location to be made on the back and the sides of the animals. Using surgical scissors and forceps, a circular excision (500 mm^2^) was formed on the shaved dorsal skin up to a certain depth (1.5 mm) of loose subcutaneous tissue. Day 0 was calculated when a circular excision wound was deemed created. Treatment was administered once daily until full epithelialization was achieved.

One-third of the experimental animals were sacrificed on the seventh post-operative day. Wound granulation tissues (excluding any underlying muscle and superfluous tissue) were collected. A tissue sample was taken and was treated for western blotting, connective tissue parameters and histopathological assessments, while another portion was preserved in a 4% formaldehyde. Following injury on day 15, half of the surviving animals were euthanized, and the retrieved tissue was utilized for histopathological analysis. The remaining animals were monitored until a complete epithelialization occurred (Upadhyay et al., 2013). For every third-day post-wounding, the perimeter of the excision wound was traced on a transparent paper until the wound closed. The wound area was measured by retracing the injury on millimeter scale graph paper and the percentage of wound contraction was estimated using the following formula:

$$Percentage\;wound\;contraction=\frac{\left(Initial\;wound\;size–\text{wound size on a specific day }\right)}{Initial\;wound\;size}\;\times \;100$$

The wound was monitored for the presence of complete epithelialization. Basically, it was measured from wounding day (baseline) until the eschar separated itself from the raw wound (Nayak et al., 2007; Dwivedi et al., 2017).

#### 2.5.2 Incision wound model

The rats were anesthetized before and throughout the wound infliction. The animals’ dorsal fur was shaved using an electric clipper. The animals were randomly divided into four groups (*n* = 6). Group I served as the control (ointment base alone), whereas Group II served as the standard (5% w/w povidone iodine ointment). Groups III and IV were given DVFO at 2.5% and 5.0% w/w, respectively. Using a sterile scalpel, a 6 cm long and 2.1 mm wide incision wound was made on the shaved skin. Following the incision, the skin was held together and sewn at 1 cm intervals of interrupted sutures using a surgical thread and a curved needle (no.8). The wounds were left naked, and the formulations were topically administered to the wound daily. On day 8, the sutures were removed, and the formulation continued to be applied. On day 10, the breaking strength of the wound was tested using Lee’s technique. Finally. the weight (in grams) required to break open the wound/skin, referred to as tensile strength, was measured (Lee, 1968; Dwivedi et al., 2017).

#### 2.5.3 Estimation of hydroxyproline content

The extracted skin tissues were dried until they reach a constant weight in a hot air oven (60°C–70°C). Subsequently, the dry tissues were hydrolyzed by 6 N HCl at 130°C for 4 h in sealed glass vials. After neutralizing the acidic tissue hydrolysate to pH 7.0 with 0.1 N potassium hydroxide, it was exposed to chloramine-T oxidation for 20 min. The reaction was stopped by adding 0.4 M perchloric acid. Subsequently, color was produced at 60°C using an Ehrlich’s reagent. A UV visible spectrophotometer was used to detect the absorbance at 557 nm. The standard calibration curve for pure 4-hydroxy-L-proline was plotted and utilized to estimate the sample concentrations in the test samples (Murthy et al., 2013).

#### 2.5.4 Estimation of hexosamine content

The hydrolyzed fraction (0.05 ml) was diluted to 0.5 ml with distilled water. Subsequently, 0.5 ml of acetylacetone reagent was added. The step was followed by heating in a boiling water bath for 20 min before cooling. Then, 1.5 ml of 96% alcohol was added, followed by the addition of 0.5 ml of Ehrlich’s reagent. The reaction was left for 30 min before measurement of color intensity at 530 nm against a blank. The hexosamine content of the samples was calculated using a standard curve produced with D (+) glucosamine hydrochloride at 5–50 μg/0.5 ml (Dische and Borenfreund., 1950).

#### 2.5.5 Histological examinations of the skin tissue

On the 7th and 15th days of the study, cross-sectional full-thickness skin specimens from each group were obtained to examine for histopathological changes. The samples were fixed in a 10% buffered formalin, processed, and blocked with paraffin before being cut into 5 μm sections. Hematoxylin and Eosin (HE) and Masson’s Trichome (MT) stains were used to stain the sections. The regenerated tissues were examined qualitatively for keratinization, epithelization, inflammation, fibroblastic proliferation, collagen depositions and neovascularization under a light microscope.

#### 2.5.6 Western blot analysis

The protein expression of collagen type 3 (COL3A1), basic fibroblast growth factor (bFGF) and vascular endothelial growth factor (VEGF) in granulation tissue from seven-day-old wounds was determined by western blotting (Feng et al., 2012). The harvested wound tissue was homogenized in radioimmunoprecipitation assay buffer (RIPA) (50 mM Tris-HCl, 150 mM NaCl, 1 mM EDTA, 1% NP-40 and 1 mM Phenazine Metho Sulfate Fluoride PMSF; pH 7.4) and centrifuged at 10,000×g for 10 min at 4°C. Protein concentration was estimated by using a Bradford reagent.

An equal amount of protein was electrophoresed onto the 12% sodium dodecyl sulphate-polyacrylamide gel electrophoresis (SDS-PAGE) at 80 V for 45 min. The proteins were trans-blotted poly vinylidene fluoride (PVDF) membrane and incubated with COL3A1, bFGF, VEGF and β-actin primary antibodies (1:1000) overnight at 4°C, at room temperature with the corresponding secondary antibodies (1: 2000) for 1–2 h. The desired proteins were detected by a Western Max-HRP-Chromogenic detection kit and 5-Bromo-4-chloro-3^'^-indolyl phosphate p-toluidine salt-Nitro Blue Tetrazolium (BCIP-NBT) solution using β-actin as the internal control.

### 2.6 Statistical analysis

Statistical evaluations were performed with an IBM SPSS (version 23) and with Microsoft excel 2007. Results of parametric tests were expressed in terms of mean ± SD. Kruskal Wallis test, followed by the Mann-Whitney *U*-test, was used in the multivariate analyses. In both test methods, the probability value *p* < 0.05 was considered as statistically significant while *p* < 0.01 was considered as highly significant.

## 3 Results

### 3.1 Percentage yield and HPLC analysis

**FIGURE 3:**
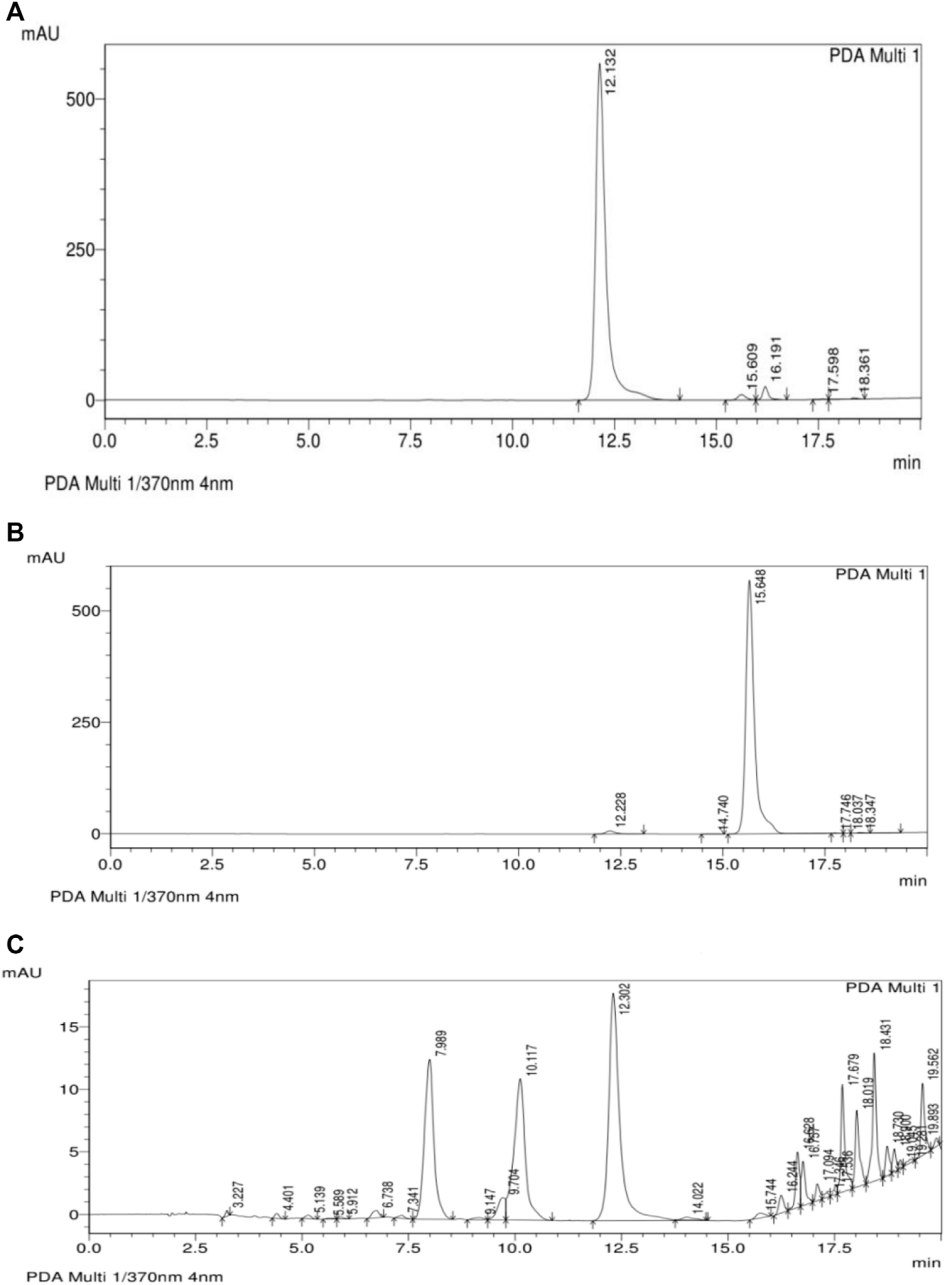
HPLC analysis of ethyl acetate fraction of **(A)** Quercetin, **(B)** Kaempferol and **(C)** Ethyl acetate fraction of *D. viscosa.*

The ethanol extract of *D. viscosa* was fractionated with ethyl acetate solvent to yield an ethyl acetate fraction (11.3%w/w) rich in flavonoids. The fraction was greenish brown semi-solid mass which was soluble in water. The flavonoids in the ethyl acetate fraction were estimated by HPLC using quercetin and kaempferol as standards. The flavonoids were identified by comparing the retention time in HPLC chromatograms of the fraction with the standards run in Similar condition. The retention time was 12.3 min (quercetin) and 15.7 min (kaempferol). The amounts of flavonoids were calculated from the peak area of the HPLC chromatogram of ethyl acetate fraction (Figure 3). Quercetin and kaempferol were determined to be 6.46% w/w and 0.132% w/w, respectively.

### 3.2 Acute dermal toxicity study

The acute toxicity study and skin irritation tests were performed as per the OECD 402 guideline to determine the formulation’s safety. A 5% w/w DVFO was applied once to the bare skin portion at the back of the rat and was observed for 14 days for an abnormal skin response including itching, redness, irritation, and other related symptoms. No evidence of toxicity with respect to clinical signs, remarkable bodyweight observations, or mortality, were recorded in all the experimental animals. The findings revealed that 5% w/w DVFO was deemed suitable and safe for further studies.

### 3.3 The effect of DVFO on wound contraction

The percentage wound contraction findings based on the excision wound model experiment following topical administration of ethyl acetate fraction ointment at different concentrations (2.5% w/w and 5.0% w/w) are shown in Table 2. The control group exhibited a contraction of 73.92% on day 15 post-operation. Nevertheless, the 2.5% w/w and 5.0% w/w DVFO-treated groups demonstrated significantly higher wound contraction than the control group on day 15 (88.88% and 94.89% respectively). Moreover, the 5% w/w DVFO-treated groups exhibited a similar percentage of wound contraction as the standard povidone-iodine group (99.66%).

**TABLE 2:** The effect of DVFO on the percentage wound contraction in excision wound.

Groups	Day 3	Day 6	Day 9	Day 12	Day 15	Epithelialization period (days)
Control	14.72 ± 0.65	34.55 ± 1.44	58.41 ± 1.32	68.49 ± 1.45	73.92 ± 1.51	21.15 ± 0.07
Standard (Povidone Iodine ointment 5%)	23.07 ± 1.22**	68.29 ± 2.44**	83.42 ± 1.24**	92.56 ± 4.51**	99.66 ± 0.19**	15.33 ± 0.04**
DVFO 2.5% w/w	18.68 ± 0.40**^b^	55.57 ± 3.45**^a^	72.27 ± 0.99**^b^	81.40 ± 0.93**^b^	88.88 ± 0.99**^b^	18.09 ± 0.09**^a^
DVFO 5% w/w	21.86 ± 0.80**	60.27 ± 0.49**^a^	80.82 ± 0.64**	89.96 ± 1.15**	94.89 ± 1.08**^a^	17.04 ± 0.11**

Values are expressed as Mean ± SD., The statistical analysis was conducted by Kruskal–Wallis test followed by Mann-Whitney *U* test. Statistical significance of differences: *p < 0.05, **p < 0.01 vs. Control and ^a^ *p* < 0.05, ^b^ *p* < 0.01 vs. Povidone iodine-treated group.

A full-thickness skin defect was induced by skin excision and the various groups were treated with base, povidone-iodine ointment and DVFO ointment at different concentrations until complete epithelialization. Wound healing, as indicated by the formation of granular tissue, occurred on day 18 in the 2.5% w/w DVFO group and on day 17 in the 5% w/w DVFO group. The data indicated that DVFO formulations significantly promote cutaneous excision wound healing in a dose-dependent manner compared to the control group (*p* < 0.01).

### 3.4 The effect of DVFO on hydroxyproline concentrations

**FIGURE 4:**
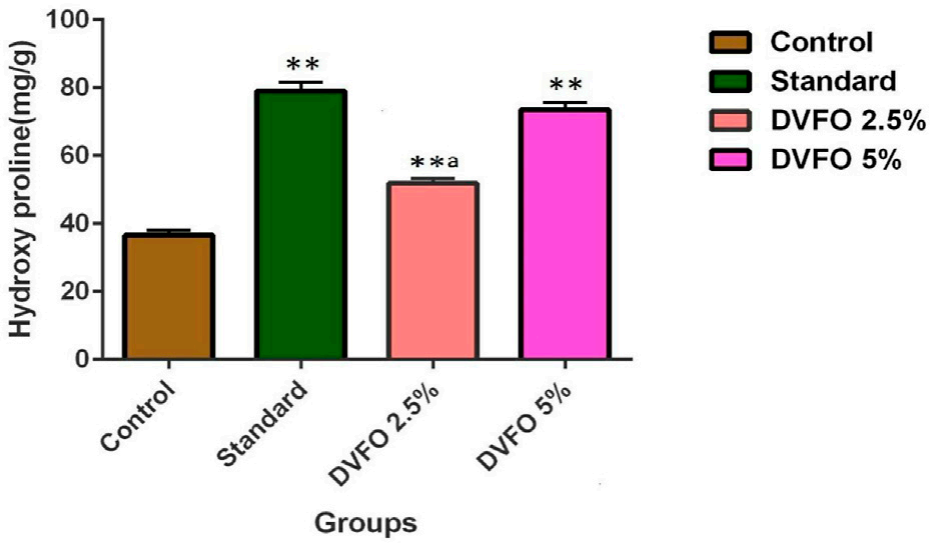
Effect of DVFO on hydroxyproline content. Values are expressed as Mean ± SD. Statistical analysis was done by Kruskal–Wallis followed by Mann-Whitney *U* test. Statistical significance: **p < 0.01 vs. Control, ^a^ *p* < 0.05 vs. Povidone iodine-treated groups.

Hydroxyproline levels in granuloma tissue treated with *D. viscosa* ethyl acetate fraction is represented in Figure 4. In the present study, the topical application of DVFO (2.5 and 5.0%w/w) significantly increased (*p* < 0.01) hydroxyproline concentrations when compared to the control group. Similarly, DVFO 5% w/w treatment (73.50 ± 2.24 mg/g) had a similar effect as povidone-iodine treated group (79.00 ± 2.63 mg/g) with respect to the hydroxyproline content indicating the wound healing potential of DVFO.

### 3.5 The effect of DVFO on hexosamine concentrations

**FIGURE 5:**
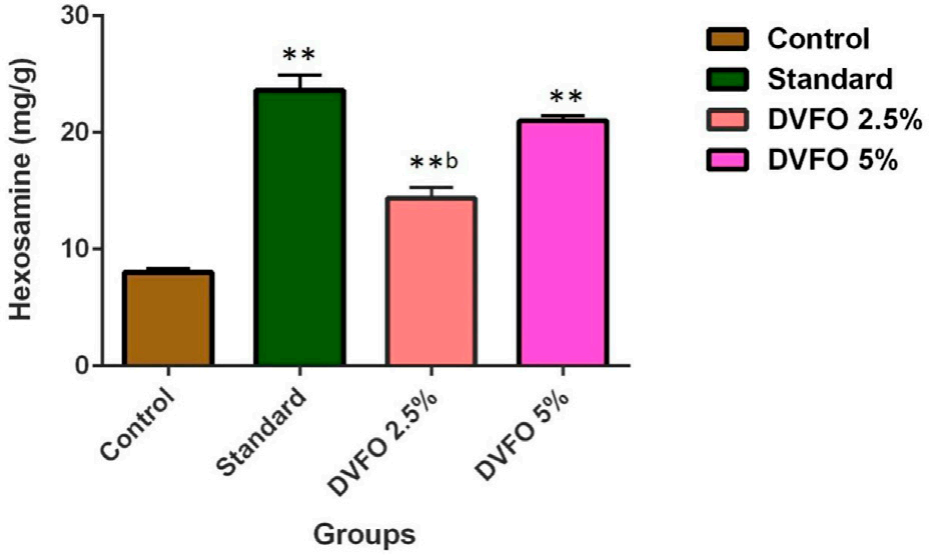
Effect of DVFO on hexosamine content. Values are expressed as Mean ± SD. Statistical analysis was done by Kruskal–Wallis followed by Mann-Whitney *U* test. Statistical significance: **p < 0.01 vs. Control, ^b^ *p* < 0.01 vs. Povidone iodine-treated groups.

Hexosamine is one of the components of glycosaminoglycans. The levels of hexosamine were significantly (*p* < 0.01) increased in 2.5% and 5.0% w/w DVFO-treated animals (14.35 ± 0.89 and 20.97 ± 0.44 mg/g, respectively) as compared to the control (8.01 ± 0.15 mg/g) (Figure 5) indicating better stabilization of collagen fibers in DVFO-treated animals.

### 3.6 The effect of DVFO on tensile strength

**FIGURE 6:**
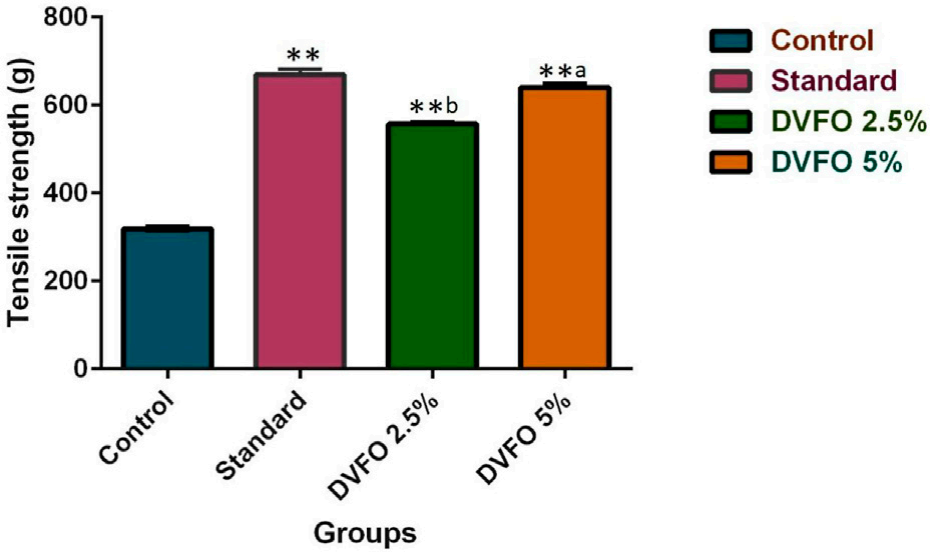
Effect of DVFO on Tensile strength. Values are expressed as Mean ± SD. Statistical analysis was done by Kruskal–Wallis followed by Mann-Whitney *U* test. Statistical significance: **p < 0.01 vs. Control, ^a^ *p* < 0.05, ^b^ *p* < 0.01 vs. Povidone iodine-treated groups.

Tensile strength is a crucial characteristic in an incision wound model (Figure 6). The minimum tensile strength of the wound in the control group was 319.28 ± 2.59 g. In comparison the mean strength of the animals from the DVFO group increased considerably in a dose-dependent manner. The mean tensile strength of a wound treated with 2.5% w/w DVFO was 557.23 ± 4.79 g, whereas a wound treated with 5% w/w DVFO had 640.48 ± 8.89 g. Tensile strengths in the DVFO-treated groups were substantially (*p* < 0.01) higher than that reported for the control group and were almost equal to those in the conventional drug-treated group (669.59 ± 12.42 g).

### 3.7 Histological analysis of skin tissues

**FIGURE 7:**
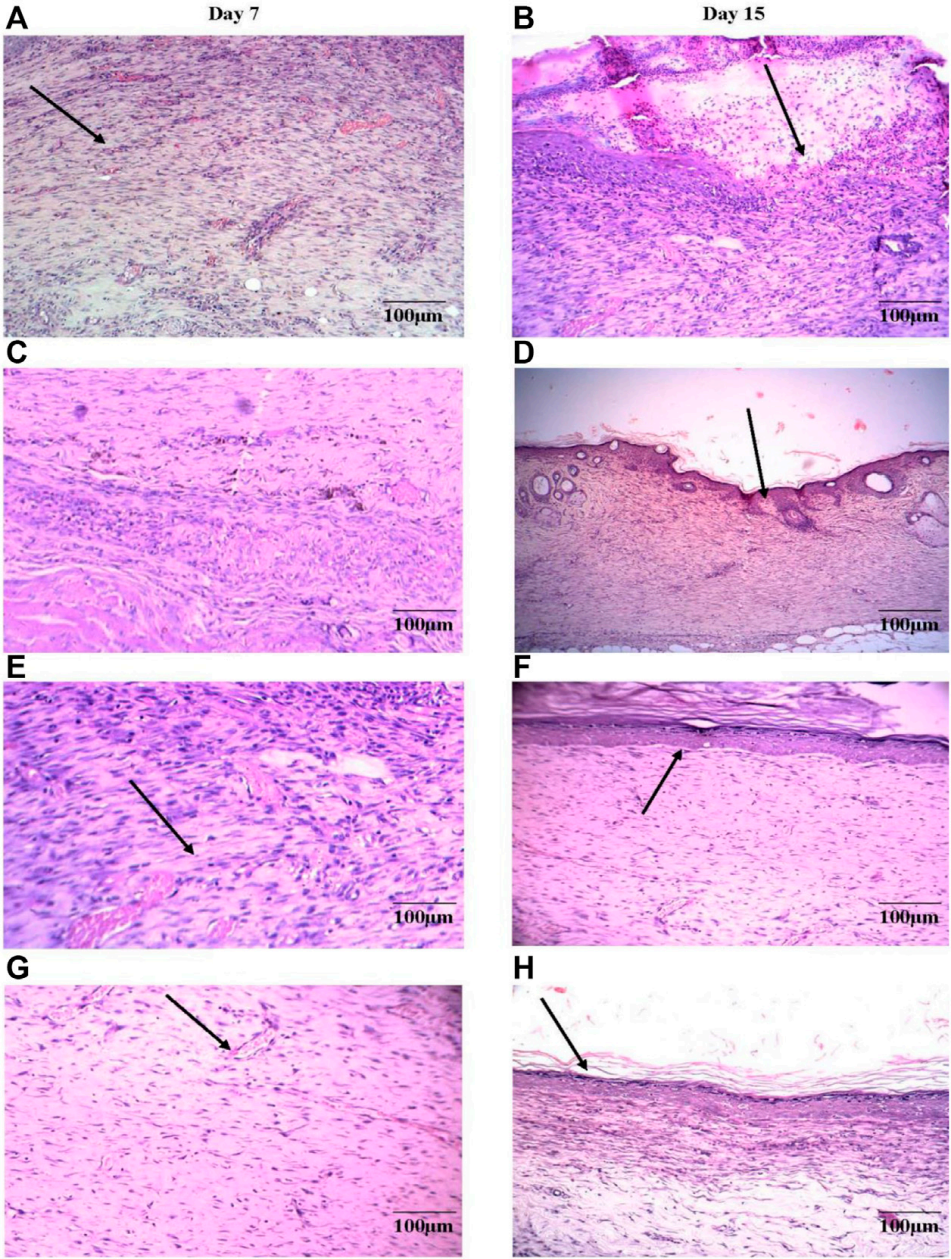
Histological evaluation of 7^th^ and 15^th^ postoperative day wound skin sections stained with hematoxylin and eosin (Bar = 100 μm). Control group [**(A)** on 7^th^ Day and **(B)** on 15^th^ Day], Standard treatment group [**(C)** on 7^th^ Day and **(D)** on 15^th^ Day], 2.5% w/w DVFO treatment group [**(E)** on 7^th^ Day and **(F)** on 15^th^ Day], 5% w/w DVFO treatment group [**(G)** on 7^th^ Day and **(H)** on 15^th^ Day].

Histopathology of skin samples was performed with hematoxylin and eosin (H&E) staining and was evaluated for changes. In the DVFO- and povidone-iodine treated groups, the histopathological sections on day 7 post-operative wound tissue revealed more significant infiltration of macrophages/fibroblasts with reduced inflammatory cells and ulcer-oedematous regions. In contrast, the control groups had moderate edema with high polymorphonuclear (inflammatory) cell density and fewer macrophages/fibroblasts. On day 7, many new capillaries and fibroblasts were seen in the granulation tissue in the DVFO- and standard treated groups, which were more prevalent than in the control group (Figure 7) indicating that DVFO stimulates neovascularization while also increasing fibroblast proliferation and migration.

**FIGURE 8:**
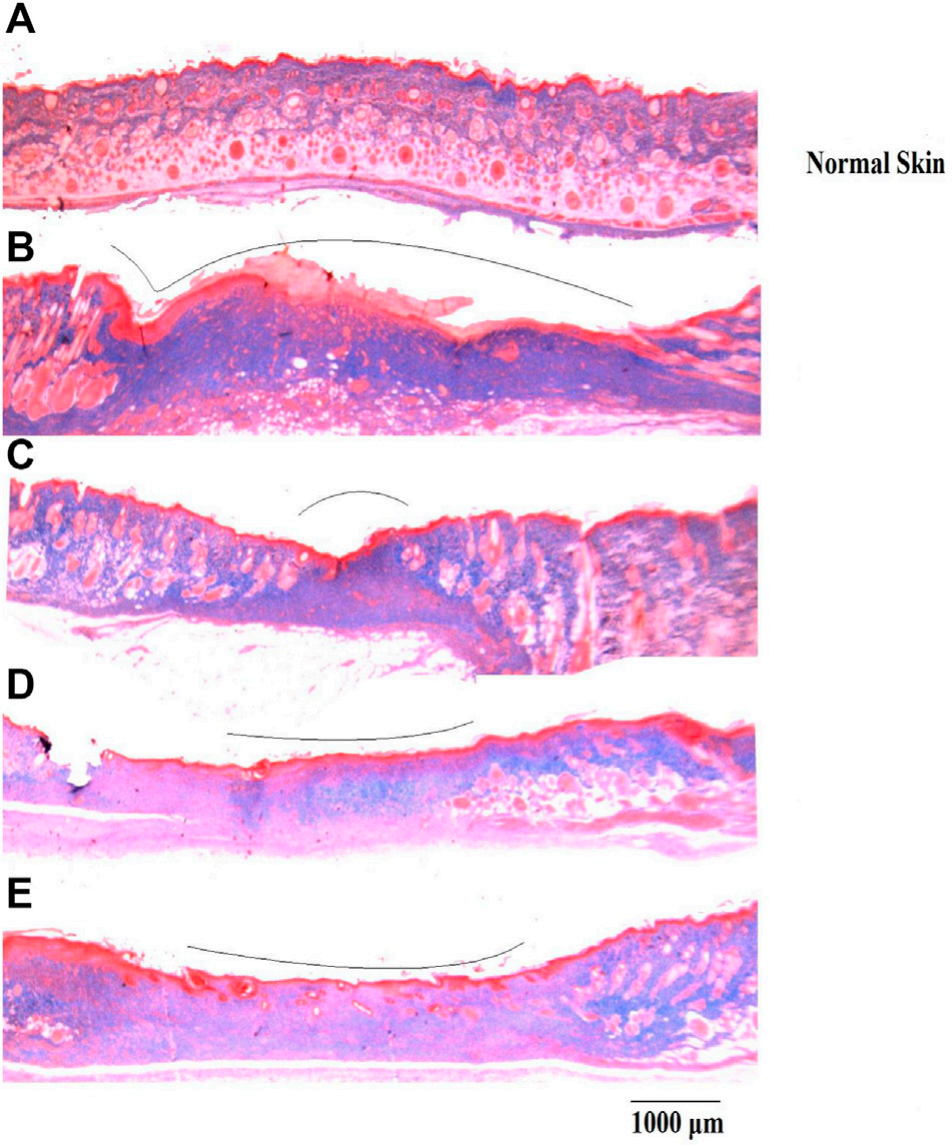
Histological evaluation of wound skin sections stained with Masson’s trichome (Bar = 1000 μm). **(A)** Normal skin, **(B)** Wound control group, **(C)** Standard treatment group, **(D)** 2.5% w/w DVFO treatment group, **(E)** 5% w/w DVFO treatment group.

On day 15, wound healing processes were well-organized following treatment with either DVFO or povidone-iodine. Wounds from the DVFO 5% (w/w) treated group had more collagen fibers and strong dermal blood vessel development with dense and thick mesenchymal matrix deposition in H&E-stained sections as compared to the control group which had fewer inflammatory cells, inadequate collagen fiber synthesis and incomplete epidermis (Figure 7). Additionally, all treated tissues lacked fibrinoid necrosis. The histology of an excision biopsy of a skin wound on day 15 revealed healed skin structures with normal epithelialization, adnexal restoration, and extensive fibrosis within the dermis in the DVFO and standard treated groups. In contrast, the control groups lag behind the treated groups in forming ground substances in the granulation tissue, as observed in the tissue sections. The DVFO- and standard treatment groups had full epithelialization, while the control group had partial epithelialization (Figure 7). Masson’s trichrome (MT) staining revealed the existence of compact collagen fibers in both the DVFO- and control groups. In fact, in the control group, collagen bundles were loosely packed, and wounds were only mildly cellular with fibroblasts (Figure 8).

### 3.8 Western blotting

**FIGURE 9:**
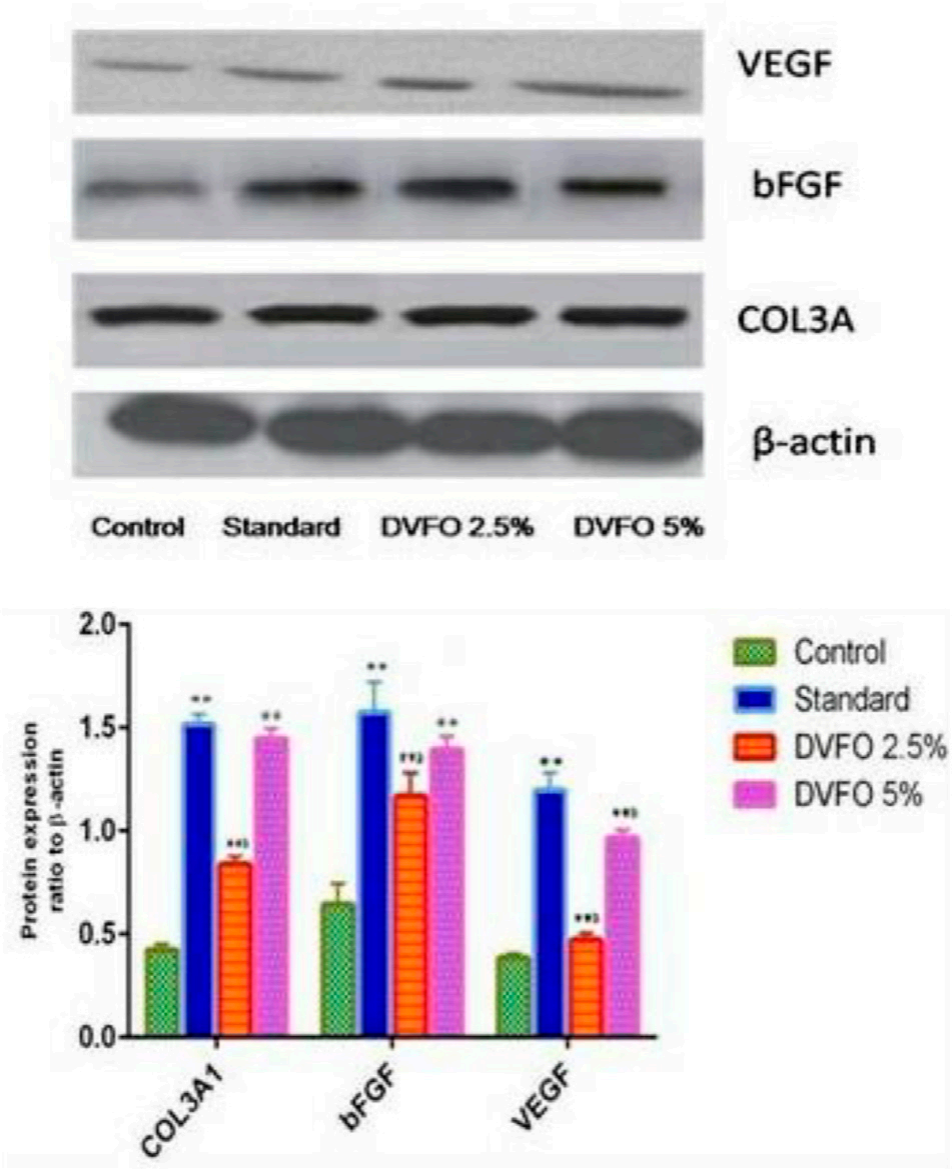
Western blot analysis of important growth factors involved in the regulation of wound healing and regeneration. The skin tissue of animals treated with DVFO showed increased expression of FGF, VEGF and COL3A as compared to control. Values are expressed as Mean ± SEM. Statistical analysis was done by Kruskal–Wallis followed by Mann-Whitney *U* test. Statistical significance: **p < 0.01 vs. Control, ^a^ *p* < 0.05, ^b^ *p* < 0.01 vs. Povidone iodine-treated groups.

Western blotting was used to examine COL3A, VEGF and bFGF protein expression levels (Figure 9). Compared to the control group, administration of DVFO substantially increased (*p* = 0.01) the COL3A, VEGF and bFGF protein expressions in the wound granulation tissue. The protein expression of COL3A, VEGF and bFGF in the standard group was considerably more significant than in the control group. Notably, COL3A, VEGF and bFGF levels in the 5% w/w DVFO- treated group were comparable to those in the control group. Overall, our findings suggest that DVFO promotes wound healing by increasing the protein expression levels of COL3A, VEGF and bFGF.

## 4 Discussion

Our work provides the scientific data to validate the traditional claims that *D. viscosa* leaves have wound-healing effects, possibly by increasing the protein expression levels of COL3A, VEGF and bFGF. Wound healing is considered a complicated and dynamic process that typically encompasses several phases signifying healing stages and necessitates the involvement of many cell types in various cellular activities (Lopez-Jornet et al., 2014). Hemostasis is the first stage of wound healing, followed by inflammation, proliferative and maturation phases. Many growth factors, cytokines, and chemokines are involved in the signalling network that controls and executes this intricate process. The TGF-beta, FGF, VEGF, Granulocyte Macrophage Colony Stimulating Factor (GM-CSF), PDGF, Connective Tissue Growth Factor (CTGF), Interleukin (IL) family, and TNF-α families are of particular significance (Barrientos et al., 2008). Failure to progress through normal stages of phases of wound healing can ultimately result in delayed wound healing. Several factors contributing to the delayed healing are diabetes, metabolic disorders, infection, venous disease, deficiencies, and age. Most existing medications used to treat wounds are costly, and some of them cause allergic responses and drug resistance (Prasad and Dorle, 2006). Plants or their phytocompounds may impact some of these wound healing stages by speeding up the healing process. Herbal remedies have generated much interest for the treatment of wounds as they are affordable and safe. (Maver et al., 2015). Thus, the potential for obtaining affordable medicines from traditional plant-based medicine has been investigated (Phan et al., 2000).

Flavonoid rich fraction of many medicinal plants such as *Tephrosia purpurea* (L.) Pers., *Martynia annua* L., *Ononidis radix* L., and *Eugenia pruniformis* Cambess (Lodhi et al., 2013; Lodhi and Singhai, 2013; de Albuquerque et al., 2016; Ergene Öz et al., 2018) have been proved to have potent wound healing efficiency. In line with this, earlier reports indicated that the plant *D. viscosa* possesses rich polyphenolic components, mainly flavonoids which are well known for their significant wound healing properties. Flavonoids such as, Quercetin, kaempferol, 3,3′,4′,5,7-Pentahydroxy flavane, 5-Hydroxy-7,3′4′-trimethoxy-6-acetoxy-3-prenylflavone, 3,4′-Dimethoxy-5,7-dihydroxyflavone, 3,5,7-Trihydroxy-4′-methoxyflavone, Pinocembrin and Penduletin has been identified from the plant (Al-Asmari and Hossain, 2016; Asila and Hossain, 2018; Getie et al., 2000; Mostafa et al., 2014; Muhammad et al., 2015; Teffo et al., 2010; Van Heerden et al., 2000 **),** and also few unexplored minor components such as di and triterpenoids. The contribution of various flavonoids in wound healing is enormous. In this context, the plant *D. viscosa* has been selected for the current investigation. Moreover, several experimental studies have explored the wound healing potential of the whole extract, such as a group of researchers have disclosed the preliminary wound healing studies with ethanol extract of *D. viscosa* leaves (Joshi et al., 2003). Similarly, another study has unveiled the wound healing potential of *D. viscosa* formulation prepared with methanol and chloroform extract of leaves in experimental animals (Nayeem et al., 2021). All the previous studies have used the whole crude extract of *D. viscosa*, and no one has explored the wound healing potential of the fractions of leaves together with mechanism of action. In the previous *in-vitro* wound healing studies, the authors compared the potential of ethanol extract and flavonoid rich fraction (Ethyl acetate fraction) of *D. viscosa* by *in-vitro* cell proliferation assay on HaCaT cell line (Shanthi et al., 2015) and the results showed that flavonoid rich fraction was more effective in inducing the proliferation on HACAT keratinocytes. Hence, the potential of the flavonoid rich fraction of *D. viscosa* as topical formulation was evaluated for wound healing efficacy. Our investigation on the acute dermal toxicity investigation indicated that DVFO was safe. Our study, which employed two distinct models to test the wound healing impact of *D. viscosa* ethyl acetate fraction on various stages of wound healing, indicated that similar to conventional treatment, DVFO decreased wound size considerably as compared to the control. Accelerated wound contraction is associated with fibroblast activation, which is mediated by specialized myofibroblasts of granulated tissues (Moulin et al., 2000). Wound contraction, which pulls the wound’s margins toward its centre during the proliferative phase of healing, improves closure of the defect (Tang et al., 2007). Contraction minimizes the amount of extracellular matrix required to repair the damage and thereby shortens the healing process. In addition, by reducing the distance that migratory keratinocytes must cover, contraction promotes re-epithelization (Strodtbeck, 2001). Furthermore, if the medicine is more effective, the wound will heal quickly (Prasad and Dorle, 2006). Therefore, the significant effect of DVFO on the wound healing process in rats may be attributed to an increase in fibroblast activities, required for proper wound closure. The positive effect of DVFO treatment was further ascertained with a histopathological examination, which confirmed normal skin histology with the reappearance of intact epidermis and dermis, fibroblast and collagen deposition compared to the control group that had disrupted tissues in the dermal wound area.

The DVFO-group exhibited faster epithelialization, while the control group exhibited a partial epithelialization. Granulation tissue development over the wound section hastens re-epithelialization due to the arrangement of epithelial cells surrounding the newly formed tissue to establish a barrier between the wound area and the external environment (Esimone et al., 2008). The wound re-epithelialization cycle is mainly associated with a faster wound healing process that can be indicated by hydroxyproline level as well. Hydroxyproline concentration is a critical component of the collagen fiber triple helix (Shoulders and Raines., 2009). An increase in hydroxyproline concentration suggests a decrease in cellular proliferation and collagen formation. In addition, hexosamine levels increase in the early phases of wound healing, indicating that fibroblasts are actively synthesizing ground material (mucopolysaccharides) on which collagen may be deposited (Ross and Benditt, 1964). In the excision wound model, topical administration of DVFO (2.5 and 5.0%w/w) significantly increased hydroxyproline, hexosamine concentrations as well as collagen formation indicating that the plant has wound-healing properties.

Tensile strength is an essential indicator of the quality and amount of epithelialized collagen as well as the strength and degree of wound healing (Raghuwanshi et al., 2017). In terms of wound healing tissue tensile strength, DVFO has an excellent wound healing property. The improved tensile strength of the treated incision wounds may be related to increased collagen content and fiber stability. Collagen, a component of developing cells, is synthesized by tissue going for repair. Collagen molecules are generated and laid down at the wound site, where they crosslink to create fibers. Wound strength is gained by both collagen remodeling and the creation of stable intra- and intermolecular crosslinks in the fibers. Consequently, wound healing effects of DVFO (2.5%and 5.0% w/w) may be linked to the synthesis and organization of newly synthesized subdermal collagen fibers as well, since there was a higher tensile strength as compared to the control group.

On day 15 after the injury, histopathological sections of both DVFO and positive control revealed newly formed collagen aligned with just a few cells. Additionally, wound healing and cell differentiation were also seen. The control wound tissue had low inflammatory cells and high fibroblasts producing the epidermal layer, with the new collagen seemingly disordered. The histopathology of wound granulation tissue demonstrated a faster wound closure rate and increased hydroxyproline content in the DVFO-treated group, which corroborated with prior findings on wound healing processes in dogs (El-Tookhy et al., 2017). The histopathological findings backed with the biochemical results showed a considerable increase in collagen and fibroblastic deposition in DVFO and standard drug treated rats compared to control animals.

The wound healing proliferation phase includes the formation and vascularization of granulation tissue, collagen production and subsequent maturation. Collagen type III is present in remodeling wound tissues and is generated by proliferating juvenile fibroblasts in response to growth factors such as basic fibroblast growth factor (bFGF) and transforming growth factor-β (TGF-β) (Ganeshkumar et al., 2012). The angiogenic molecule bFGF increases neovascularization and blood flow to granulation tissue. Increased oxygen supply promotes collagen fiber development in granulation tissue (Kondo and Ishida, 2010). VEGF increases angiogenesis/vasculogenesis, vascular permeability, endothelial cell proliferation and migration and leukocyte adherence. Further studies indicated that VEGF increases endothelial cell hydrogen sulfide generation and release, resulting in endothelial cell proliferation, migration and permeability, micro vessel formation and wound healing. Furthermore, VEGF stimulates wound epithelialization and collagen deposition (Bao et al., 2009).

The growth factors bFGF and VEGF have been known to play an important role in angiogenesis. Angiogenesis, or the formation of new blood vessels, is involved in various physiological and pathological processes, including ovulation, embryogenesis, wound healing, inflammation, malignant tumor development, retinopathies, rheumatoid arthritis, and angiogenesis-dependent disorders (Bitto et al., 2008). In the DVFO treated group, both FGF and VEGF were up-regulated. Increased expression of growth factors was identified in the DVFO-treated group in the current investigation, showing that ethyl acetate fraction increases angiogenesis and collagen formation and mediates the wound healing process. The wound healing effect of Quercetin from *Oxytropis falcata* Bunge in cutaneous wound healing in mice was demonstrated by Mi and co-authors, reduced the inflammatory factors (Tumor Necrosis Factor-α, IL-1β and IL-6), increased expression levels of VEGF, FGF and alpha smooth muscle actin, abundant collagen formation, improved levels of antioxidant enzymes and act viaWnt/β-catenin signaling pathway (Mi et al., 2022). Our results are in good agreement with the studies of Mi and co-workers, evidence that flavonoids excerts the wound healing effect by stimulating VEGF and FGF.

The phytochemical research indicated that the ethyl acetate fraction of *D. viscosa* leaves contain flavonoids, which have also been shown to have anti-bacterial and free radical scavenging activities (Panche et al., 2016). Quercetin and kaempferol which are flavonols have remarkable potentials to function as antioxidants, anti-bacterial and anti-inflammatory agents by promoting the rapid healing of injured skin tissue (Serafini et al., 2010; Gopalakrishnan et al., 2016). In addition to other phenolic compounds with free radical scavenging activities, quercetin and kaempferol can improve wound contraction rates (Gomathi et al., 2003; Ozay et al., 2019). These aids wound healing and protect tissues from oxidative injury by lowering free radical levels. It is plausible that the phenolic chemicals and flavonoids in *D. viscosa* extract acted synergistically to create a substantial dermal wound healing effect. According to Sutthammikorn et al., 2021, topical application of *Gynura procumbens* (Lour.) Merr., contained kaempferol and quercetin compounds on the wounded skin of diabetic mice accelerated wound healing and induced the expression of angiogenin, EGF, FGF, TGF and VEGF. Wound healing potential of *Eugenia pruniformis* Cambess., hydroethanol and ethyl acetate extract were studied by De Albuquerque et al. and suggested that ethyl acetate extract was effective due to the presence of Quercetin, kaempferol and hyperoside (De Albuquerque et al., 2016). Moreover, topical administration of *Moringa oleifera* Lam., extract in excision wounds exhibited the inhibitory potential of pro-inflammatory cytokine IL-6 and endopeptidase MMPs (matrix metalloproteinases) subtype I and II, which are attributed to the flavonoid constituents *viz.* Quercetin, caffeic acid, and kaempferol (Shady et al., 2022). Previous reports indicate that the efficacy of plant extracts rich in flavonoids such as Quercetin and Kaempferol attenuates the wound healing *via* reducing the inflammatory markers, increasing the expression of growth factors and abundant collagen formation which corroborates with the reports of present study with flavonoid rich fraction of *D. viscosa*.

Quercetin and Kaempferol are reported to possess potent antioxidant ability by scavenging free radicals. Hanasaki et al. proved that Quercetin is the most potent free radical scavenger among the flavonoids (Hanasaki et al., 1994). It exhibits antioxidant effect by directly scavenging free radicals (Oh et al., 2019), by Chelating metal ions (Tang et al., 2014), by Inhibiting lipid peroxidation (Lim et al., 1998) and by regulating the antioxidant enzymes (Kobori et al., 2015). It also has a broad spectrum of antimicrobial activities (Qin et al., 2009). Both the flavonoids excerts the anti-inflammatory effect in Chang Liver cells through the inhibition of inducible nitric oxide synthase, cyclooxygenase-2 and reactive C-protein, and causes down-regulation of the nuclear factor kappaB pathway (García-Mediavilla et al., 2007). The antioxidant, anti-microbial and anti-inflammatory potential of Quercetin and Kaempferol may also support the wound healing potential of *D. viscosa.*

The present investigation confirmed the wound healing potential of flavonoid rich fraction of *D. viscosa* since DVFO at both concentrations impart considerable healing effects in excision and incision wound models when applied topically for several days. Our findings were further supported by a significant acceleration of wound contraction rate, increased in hydroxyproline concentration and reduction in the time required for granulation tissue development. Our biochemical and histopathological tests further verified the favorable impact of DVFO on wound healing. The various phytoconstituents (phenolic compounds and flavonoids) have been shown to accelerate wound healing, owing to their antioxidant, anti-microbial and anti-inflammatory characteristics. Consequently, the current investigation revealed that topical use of *D. viscosa* ethyl acetate fraction improves wound healing by stimulating collagen formation and generating up-regulation of bFGF and VEGF in wound granulation tissue that have been further supported by the histopathological findings.

**FIGURE 10:**
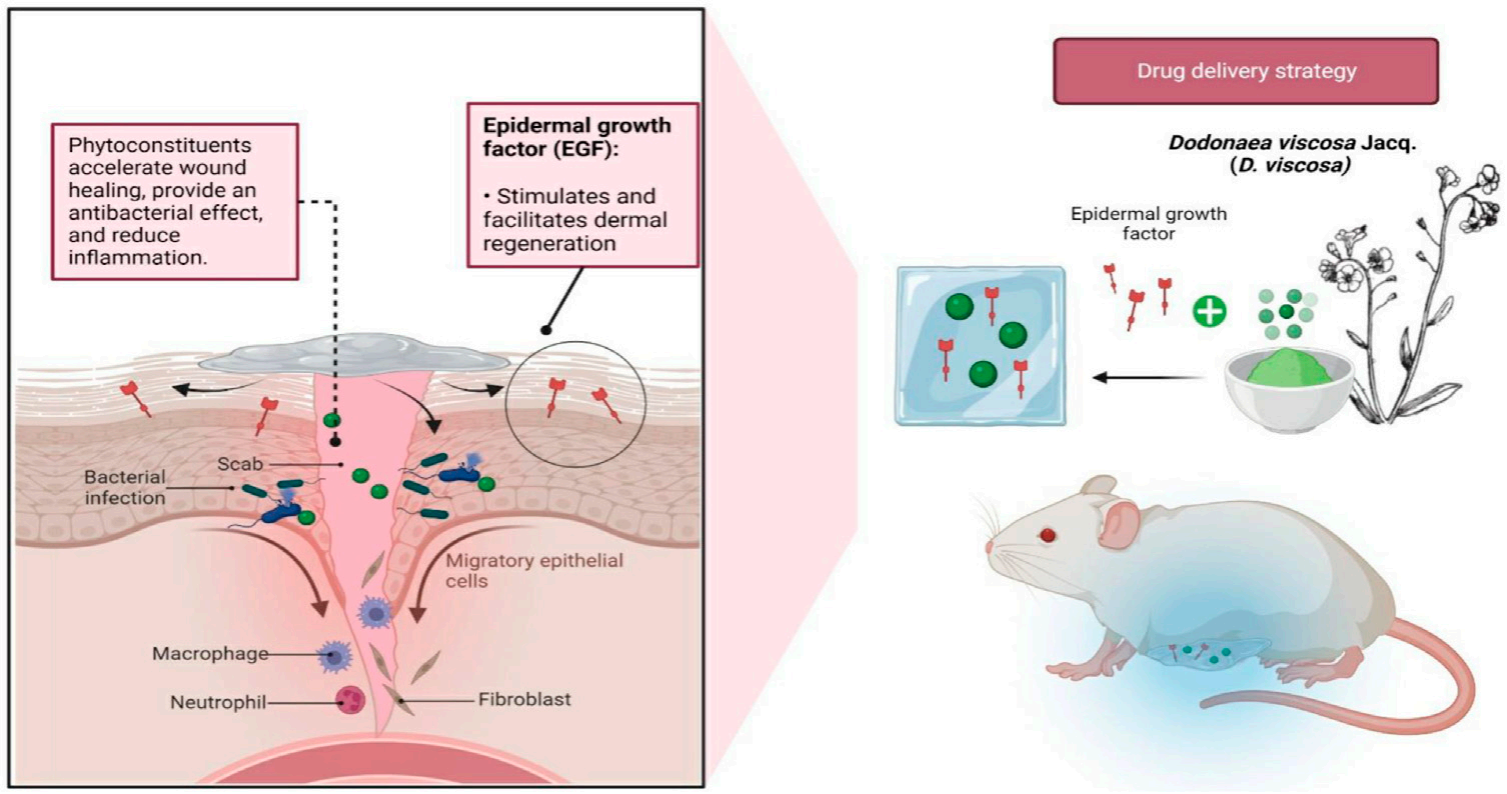
Future perspectives of encapsulating *D. viscosa* phytoconstituents into hydrogels with epidermal growth factor (EGF) against infected wounds. The flavonoids found in the plant may expedite healing and the eradication of bacteria, whereas the presence of EGF will facilitate in dermal regeneration.

As for future perspectives, nanocarriers can be implemented to encapsulate the *D. viscosa* ethyl acetate fraction for a more effective and targeted delivery of the bioactive compounds. Up until now, the plant extract has been used to biologically synthesize gold nanoparticles (Anandan and Gurumallesh Prabu, 2018), silver nanoparticles (Sebelemetja et al., 2020) and stabilised polymeric nanoparticles (PLGA-PEG) for the treatment of dental caries (Sebelemetja et al., 2020), but none have reported on loading the extract in a hydrogel embedded with epidermal growth factor (EGF). The rate of healing of a chronic wound can be accelerated by applying drugs topically at the wound site in accordance with physiological necessity (Johnson and Wang, 2015). Here, we suggest the use of hydrogel, where the polymer can be tailored to have specific properties like pH sensitivity (Hendi et al., 2020) and thermosensitivity (Huang et al., 2019; Fan et al., 2022) to achieve smart delivery strategies. In addition to the flavonoid from *D. viscosa*, which aids in bacterial eradication, EGF may benefit skin regeneration by promoting cell formation, proliferation, and differentiation (Leonard and Koria, 2017) (Figure 10). Even though loading bioactive chemicals onto nanocarriers continues to be of interest, more investigation is needed to examine the stability, drug release, and cytotoxicity of such formulations to fully comprehend their pharmacokinetic and pharmacodynamic properties.

## 5 Limitations of the study

In the present study, the effect of ethyl acetate fraction of *D. viscosa* on COL3A, VEGF and bFGF has been established; however, Influence of Inflammatory markers may be studied in future. Isolation of lead molecule from the ethyl acetate fraction also needs to be done. In future, clinical trials on flavonoid-rich ethyl acetate fraction/lead molecule of *D. viscosa* should be conducted to confirm its potential as an innovative wound healing formulation for the treatment of wounds in humans.

## 6 Conclusion

The ethyl acetate fraction of *D. viscosa* has potential wound healing activity, purported to be due to the rich flavonoids. It acts by increasing the production of COL3A, VEGF and bFGF protein in wound granulation tissue, which are significant growth factors for wound healing. Therefore, the *D. viscosa* ethyl acetate fraction, which is rich in flavonoid compounds, may have potential value as a topical medicinal treatment to accelerate wound healing.

## Data Availability

The raw data supporting the conclusion of this article will be made available by the authors, without undue reservation.

## Abbreviations

bFGF, basic fibroblast Growth Factor; COL3A, Collagen type 3; CPCSEA, committee for the purpose of control and supervision of experimental animals; DVFO, *Dodonaeaviscosa* fraction ointment; OECD, organisation for economic co-operation and development; VEGF, vascular endothelial growth factor.

## References

[B1] Al-Aamri K. K. Hossain M. A. (2016). New prenylated flavonoids from the leaves of *Dodonea viscosa* native to the Sultanate of Oman. Pac. Sci. Rev. A Nat. Sci. Eng. 18 (1), 53–61. 10.1016/j.psra.2016.08.001

[B2] Anandan M. Gurumallesh Prabu H. (2018). Dodonaea viscosa leaf extract assisted synthesis of gold nanoparticles: Characterization and cytotoxicity against A549 NSCLC cancer cells. J. Inorg. Organomet. Polym. Mater. 28 (3), 932–941. 10.1007/s10904-018-0799-6

[B3] Aruna M. Asha V. V. (2008). Gastroprotective effect of *Dodonaeaviscosa* on various experimental ulcer models. J. Ethnopharmacol. 118 (3), 460–465. 10.1016/j.jep.2008.05.026 18603387

[B4] Asila A. S. H. Hossain M. A. (2018). Isolation, structure characterization and prediction of antioxidant activity of two new compounds from the leaves of Dodonaea viscosa native to the Sultanate of Oman. Egypt. J. Basic Appl. Sci. 5, 157–164. 10.1016/j.ejbas.2018.04.004

[B5] Bao P. Kodra A. Tomic-Canic M. Golinko M. S. Ehrlich H. P. Brem H. (2009). The role of vascular endothelial growth factor in wound healing. J. Surg. Res. 153 (2), 347–358. 10.1016/j.jss.2008.04.023 19027922 PMC2728016

[B6] Barrientos S. Stojadinovic O. Golinko M. S. Brem H. Tomic-Canic M. (2008). Growth factors and cytokines in wound healing. Wound Repair Regen. 16 (5), 585–601. 10.1111/j.1524-475X.2008.00410.x 19128254

[B7] Beshah F. Hunde Y. Getachew M. Bachheti R. K. Husen A. Bachheti A. (2020). Ethnopharmacological, phytochemistry and other potential applications of Dodonaea genus: A comprehensive review. Curr. Res. Biotechnol. 2, 103–119. 10.1016/j.crbiot.2020.09.002

[B8] Bitto A. Minutoli L. Altavilla D. Polito F. Fiumara T. Marini H. (2008). Simvastatin enhances VEGF production and ameliorates impaired wound healing in experimental diabetes. Pharmacol. Res. 57 (2), 159–169. 10.1016/j.phrs.2008.01.005 18316203

[B9] Budovsky A. Yarmolinsky L. Ben‐Shabat S. (2015). Effect of medicinal plants on wound healing. Wound Repair Regen. 23 (2), 171–183. 10.1111/wrr.12274 25703533

[B10] Carvalho M. T. B. Araújo-Filho H. G. Barreto A. S. Quintans-Júnior L. J. Quintans J. S. S. Barreto R. S. S. (2021). Wound healing properties of flavonoids: A systematic review highlighting the mechanisms of action. Phytomedicine 90, 153636. 10.1016/j.phymed.2021.153636 34333340

[B11] de Albuquerque R. D. Perini J. A. Machado D. E. Angeli-Gamba T. Esteves R. D. Santos M. G. (2016). Wound healing activity and chemical standardization of Eugenia pruniformis cambess. Pharmacogn. Mag. 12 (48), 288–294. 10.4103/0973-1296.192206 27867271 PMC5096275

[B12] Dische Z. Borenfreund E. (1950). A spectrophotometric method for the microdetermination of hexosamines. J. Biol. Chem. 184 (2), 517–522. 10.1016/S0021-9258(19)50982-6 15428432

[B13] Dwivedi D. Dwivedi M. Malviya S. Singh V. (2017). Evaluation of wound healing, anti-microbial and antioxidant potential of *Pongamia pinnata* in wistar rats. J. traditional complementary Med. 7 (1), 79–85. 10.1016/j.jtcme.2015.12.002 PMC5198820 28053891

[B14] El-Tookhy O. S. Shamaa A. A. Shehab G. G. Abdallah A. N. Azzam O. M. (2017). Histological evaluation of experimentally induced critical size defect skin wounds using exosomal solution of mesenchymal stem cells derived microvesicles. Int. J. Stem Cells 10 (2), 144–153. 10.15283/ijsc17043 29084422 PMC5741195

[B15] Ergene Öz B. Saltan İşcan G. Küpeli Akkol E. Süntar İ. Bahadır Acıkara Ö. (2018). Isoflavonoids as wound healing agents from Ononidis Radix. J. Ethnopharmacol. 211, 384–393. 10.1016/j.jep.2017.09.029 28989011

[B16] Esimone C. Nworu C. Jackson C. (2008). Cutaneous wound healing activity of a herbal ointment containing the leaf extract of *Jatropha curcas* L. (Euphorbiaceae). Inter J Appl Res Nat Prod 1, 1–4.

[B17] Fan R. Cheng Y. Wang R. Zhang T. Zhang H. Li J. (2022). Thermosensitive hydrogels and advances in their application in disease therapy. Polym. (Basel) 14 (12), 2379. 10.3390/polym14122379 PMC9227257 35745954

[B18] Feng X. T. Wang T. Z. Chen Y. Liu J. B. Liu Y. Wang W. J. (2012). Pollen Typhae total flavone improves insulin-induced glucose uptake through the β-arrestin-2-mediated signaling in C2C12 myotubes. Int. J. Mol. Med. 30 (4), 914–922. 10.3892/ijmm.2012.1061 22825681

[B19] Fraga C. G. Croft K. D. Kennedy D. O. Tomás-Barberán F. A. (2019). The effects of polyphenols and other bioactives on human health. Food Funct. 10 (2), 514–528. 10.1039/c8fo01997e 30746536

[B20] Ganeshkumar M. Ponrasu T. Krithika R. Iyappan K. Gayathri V. S. Suguna L. (2012). Topical application of *Acalypha indica* accelerates rat cutaneous wound healing by up-regulating the expression of Type I and III collagen. J. Ethnopharmacol. 142 (1), 14–22. 10.1016/j.jep.2012.04.005 22521732

[B21] García-Mediavilla M. V. Crespo I. Collado P. S. Esteller A. Sánchez-Campos S. Tuñón M. J. (2007). The anti-inflammatory flavones quercetin and kaempferol cause inhibition of inducible nitric oxide synthase, cyclooxygenase-2 and reactive C-protein, and down-regulation of the nuclear factor kappaB pathway in Chang Liver cells. Eur. J. Pharmacol. 557, 221–229. 10.1016/j.ejphar.2006.11.014 17184768

[B22] Getie M. Gebre-Mariam T. Rietz R. Höhne C. Huschka C. Schmidtke M. (2003). Evaluation of the anti-microbial and antiinflammatory activities of the medicinal plants *Dodonaeaviscosa, Rumex nervosus* and *Rumex abyssinicus* . Fitoterapia 74 (1-2), 139–143. 10.1016/S0367-326X(02)00315-5 12628410

[B23] Getie M. G. Gebre-Miriam T. Rietz R. Neubert R. (2000). Distribution of quercetin, kaempferol and isorhamnetin in some Ethiopian medicinal plants used for treatment of dermatological disorders. Ethiop. Pharmacol. J. 18, 25–34.

[B24] Ghisalberti E. (1998). Ethnopharmacology and phytochemistry of *Dodonaea* species. Fitoterapia 69 (A), 99–113.

[B25] Gomathi K. Gopinath D. Rafiuddin Ahmed M. Jayakumar R. (2003). Quercetin incorporated collagen matrices for dermal wound healing processes in rat. Biomaterials 24 (16), 2767–2772. 10.1016/s0142-9612(03)00059-0 12711523

[B26] Gonzalez A. C. Costa T. F. Andrade Z. A. Medrado A. R. (2016). Wound healing - a literature review. An. Bras. Dermatol. 91 (5), 614–620. 10.1590/abd1806-4841.20164741 27828635 PMC5087220

[B27] Gopalakrishnan A. Ram M. Kumawat S. Tandan S. Kumar D. (2016). Quercetin accelerated cutaneous wound healing in rats by increasing levels of VEGF and TGF-β1. Indian J. Exp. Biol. 54 (3), 187–195. 27145632

[B28] Guo S. A. DiPietro L. A. (2010). Factors affecting wound healing. J. Dent. Res. 89 (3), 219–229. 10.1177/0022034509359125 20139336 PMC2903966

[B29] Hanasaki Y. Ogawa S. Fukui S. (1994). The correlation between active oxygens scavenging and antioxidative effects of flavonoids. Free Radic. Biol. Med. 16 (6), 845–850. 10.1016/0891-5849(94)90202-x 8070690

[B30] Hendi A. Umair Hassan M. Elsherif M. Alqattan B. Park S. Yetisen A. K. (2020). Healthcare applications of pH-sensitive hydrogel-based devices: A review. Int. J. Nanomedicine 15, 3887–3901. 10.2147/IJN.S245743 32581536 PMC7276332

[B31] Henry G. Garner W. L. (2003). Inflammatory mediators in wound healing. Surg. Clin. 83 (3), 483–507. 10.1016/S0039-6109(02)00200-1 12822721

[B32] Hsü H. Y. Chen Y. P. Kakisawa H. (1971). Structure of hautriwaic acid. Phytochemistry 10 (11), 2813–2814. 10.1016/S0031-9422(00)97286-8

[B33] Huang H. Qi X. Chen Y. Wu Z. (2019). Thermo-sensitive hydrogels for delivering biotherapeutic molecules: A review. Saudi Pharm. J. 27 (7), 990–999. 10.1016/j.jsps.2019.08.001 31997906 PMC6978621

[B34] Johnson N. R. Wang Y. (2015). Drug delivery systems for wound healing. Curr. Pharm. Biotechnol. 16 (7), 621–629. 10.2174/1389201016666150206113720 25658378 PMC6053062

[B35] Joshi S. D. Aravind M. B. Ashok K. Veerapur V. P. Shastry C. S. (2003). Wound healing activity of *Dodonaea viscosa* leaves. Indian drugs 40, 549–552.

[B36] Jucá M. M. Cysne Filho F. M. S. de Almeida J. C. Mesquita D. D. S. Barriga J. R. M. Dias K. C. F. (2020). Flavonoids: Biological activities and therapeutic potential. Nat. Prod. Res. 34 (5), 692–705. 10.1080/14786419.2018.1493588 30445839

[B37] Khalil N. M. Sperotto J. S. Manfron M. P. (2006). Antiinflammatory activity and acute toxicity of *Dodonaeaviscosa* . Fitoterapia 77 (6), 478–480. 10.1016/j.fitote.2006.06.002 16884859

[B38] Kirtikar K. R. Basu B. D. (1995). Indian medicinal plants. Allahabad, India: Lalit Mohan Publication.

[B39] Kobori M. Takahashi Y. Akimoto Y. Sakurai M. Matsunaga I. Nishimuro H. (2015). Chronic high intake of quercetin reduces oxidative stress and induces expression of the antioxidant enzymes in the liver and visceral adipose tissues in mice. J. Funct. Foods 15, 551–560. 10.1016/j.jff.2015.04.006

[B40] Kondo T. Ishida Y. (2010). Molecular pathology of wound healing. Forensic Sci. Int. 203 (1-3), 93–98. 10.1016/j.forsciint.2010.07.004 20739128

[B41] Lawrence W. T. (1998). Physiology of the acute wound. Clin. plastic Surg. 25 (3), 321–340. 10.1016/S0094-1298(20)32467-6 9696896

[B42] Lee K. H. (1968). Studies on the mechanism of action of salicylate II. Retardation of wound healing by aspirin. J. Pharm. Sci. 57 (6), 1042–1043. 10.1002/jps.2600570633 5671327

[B43] Leonard A. Koria P. (2017). Growth factor functionalized biomaterial for drug delivery and tissue regeneration. J. Bioact. Compat. Polym. 32 (6), 568–581. 10.1177/0883911517705403 29062166 PMC5649639

[B44] Lim B. Yu B. Cho S. Her E. Park D. (1998). The inhibition by quercetin and ganhuangenin on oxidatively modified low density lipoprotein. Phytotherapy Res. 12 (5), 340–345. 10.1002/(SICI)1099-1573(199808)12:5<340:AID-PTR316>3.0.CO;2-U

[B45] Lodhi S. Jain A. Sharma V. K. Singhai A. K. (2013). Wound-healing effect of flavonoid-rich fraction from *Tephrosia purpurea* linn. On streptozotocin-induced diabetic rats. J. Herbs, Spices Med. Plants 19 (2), 191–205. 10.1080/10496475.2013.779620

[B46] Lodhi S. Singhai A. K. (2013). Wound healing effect of flavonoid rich fraction and luteolin isolated from Martynia annua Linn. on streptozotocin induced diabetic rats. Asian Pac. J. Trop. Med. 6 (4), 253–259. 10.1016/S1995-7645(13)60053-X 23608325

[B47] Lopez‐Jornet P. Camacho‐Alonso F. Gómez‐Garcia F. Molina Minano F. Cañas X. Serafín A. (2014). Effects of potassium apigenin and verbena extract on the wound healing process of SKH‐1 mouse skin. Int. wound J. 11 (5), 489–495. 10.1111/j.1742-481X.2012.01114.x 23136845 PMC7950762

[B48] Mashelkar R. A. (2008). Wealth of India, first supplement series (raw materials). New Delhi: Council of Scientific and Industrial Research.

[B49] Maver T. Maver U. StanaKleinschek K. Smrke D. M. Kreft S. (2015). A review of herbal medicines in wound healing. Int. J. dermatology 54 (7), 740–751. 10.1111/ijd.12766 25808157

[B50] Mi Y. Zhong L. Lu S. Hu P. Pan Y. Ma X. (2022). Quercetin promotes cutaneous wound healing in mice through Wnt/β-catenin signaling pathway. J. Ethnopharmacol. 290, 115066. 10.1016/j.jep.2022.115066 35122975

[B51] Mohd Zaid N. A. Sekar M. Bonam S. R. Gan S. H. Lum P. T. Begum M. Y. (2022). Promising natural products in new drug design, development and therapy for skin disorders: An overview of scientific evidence and understanding their mechanism of action. Drug Des. DevelTher 16, 23–66. 10.2147/DDDT.S326332 PMC8749048 35027818

[B52] Mostafa A. E. El-hela A. A. Mohammad A. I. Jacob M. Cutler S. J. Ross S. A. (2014). New secondary metabolites from Dodonaea viscosa. Phytochem. Lett. 8, 10–15. 10.1016/j.phytol.2013.12.008

[B53] Moulin V. Auger F. A. Garrel D. Germain L. (2000). Role of wound healing myofibroblasts on re-epithelialization of human skin. Burns 26 (1), 3–12. 10.1016/S0305-4179(99)00091-1 10630313

[B54] Muhammad A. Tel-cayan G. Öztürk M. Nadeem S. Duru M. E. Anis I. (2015). Biologically active flavonoids from Dodonaea viscosa and their structure activity relationships. Industrial Crops Prod. 78, 66–72. 10.1016/j.indcrop.2015.10.011

[B55] Murthy S. Gautam M. K. Goel S. Purohit V. Sharma H. Goel R. K. (2013). Evaluation of *in-vivo* wound healing activity of *Bacopa monniera* on different wound model in rats. BioMed Res. Int. 2013, 972028. 10.1155/2013/972028 23984424 PMC3745907

[B56] Nayak B. S. Anderson M. Pereira L. P. (2007). Evaluation of wound-healing potential of *Catharanthus roseus* leaf extract in rats. Fitoterapia 78 (7-8), 540–544. 10.1016/j.fitote.2007.06.008 17683880

[B57] Nayeem N. Asdaq S. M. B. Alamri A. S. Alsanie W. F. Alhomranic M. Mohzari Y. (2021). Wound healing potential of Dodonaea viscosa extract formulation in experimental animals. J. King Saud Univ. – Sci. 33 (5), 101476. 10.1016/j.jksus.2021.101476

[B58] NourianDehkordi A. MirahmadiBabaheydari F. Chehelgerdi M. Raeisi Dehkordi S. (2019). Skin tissue engineering: Wound healing based on stem-cell-based therapeutic strategies. Stem Cell Res. Ther. 10, 111. 10.1186/s13287-019-1212-2 30922387 PMC6440165

[B59] OECD (1987). Guidelines for testing of chemicals. Acute Dermal Toxic. 402, 1–7.

[B60] Oh W. Y. Ambigaipalan P. Shahidi F. (2019). Preparation of quercetin esters and their antioxidant activity. J. Agric. food Chem. 67 (38), 10653–10659. 10.1021/acs.jafc.9b04154 31464427

[B61] Oliveira B. H. D. Nakashima T. Souza Filho J. D. D. Frehse F. L. (2001). HPLC analysis of flavonoids in *Eupatorium littorale* . J. Braz. Chem. Soc. 12, 243–246. 10.1590/S0103-50532001000200019

[B62] Ozay Y. Güzel S. Yumrutaş Ö. Pehlivanoğlu B. Erdoğdu İ. H. Yildirim Z. (2019). Wound healing effect of kaempferol in diabetic and nondiabetic rats. J. Surg. Res. 233, 284–296. 10.1016/j.jss.2018.08.009 30502261

[B63] Panche A. N. Diwan A. D. Chandra S. R. (2016). Flavonoids: An overview. J. Nutr. Sci. 5, e47. 10.1017/jns.2016.41 28620474 PMC5465813

[B64] Phan T. T. Lee S. T. Chan S. Y. Huge M. A. Cherry A. W. (2000). Investigation plant based medicines for wound healing with the use of cell culture technologies and *in-vitro* models. A review. Annu. Acad. Med. Singap. 29 (1), 27–36. 10748961

[B65] Polerà N. Badolato M. Perri F. Carullo G. Aiello F. (2019). Quercetin and its natural sources in wound healing management. Curr. Med. Chem. 26 (31), 5825–5848. 10.2174/0929867325666180713150626 30009700

[B66] Prasad V. Dorle A. K. (2006). Evaluation of ghee based formulation for wound healing activity. J. Ethnopharmacol. 107 (1), 38–47. 10.1016/j.jep.2006.02.006 16546334

[B67] Qin X. R. Zhang M. J. Gao X. N. Lin Y. Li M. A. Si-Yi H. E. (2009). Study on the antibacterial activity of quercetin. Chem. Bioeng. 26 (4), 55–57.

[B68] Raghuwanshi N. Kumari P. Srivastava A. K. Vashisth P. Yadav T. C. Prasad R. (2017). Synergistic effects of *Woodfordiafruticosa* gold nanoparticles in preventing microbial adhesion and accelerating wound healing in Wistar albino rats *in-vivo* . Mater. Sci. Eng. C 80, 252–262. 10.1016/j.msec.2017.05.134 28866163

[B69] Richardson M. (2004). Acute wounds: An overview of the physiological healing process. Nurs. times 100 (4), 50–53. 14974265

[B70] Rodrigues M. Kosaric N. Bonham C. A. Gurtner G. C. (2019). Wound healing: A cellular perspective. Physiol. Rev. 99 (1), 665–706. 10.1152/physrev.00067.2017 30475656 PMC6442927

[B71] Ross R. Benditt E. P. (1964). Wound healing and collagen formation: IV. Distortion of ribosomal patterns of fibroblasts in scurvy. J. Cell Biol. 22 (2), 365–389. 10.1083/jcb.22.2.365 14203386 PMC2106449

[B72] Sachdev K. Kulshreshtha D. K. (1983). Flavonoids from *dodonaeaviscosa* . Phytochemistry 22 (5), 1253–1256. 10.1016/0031-9422(83)80234-9

[B73] Saini N. Gahlawat S. K. Lather V. (2017). “Flavonoids: A nutraceutical and its role as anti-inflammatory and anticancer agent,” in Plant biotechnology: Recent advancements and developments. Editors Gahlawat S. Salar R. Siwach P. Duhan J. Kumar S. Kaur P. (Singapore: Springer).

[B74] Sebelemetja M. Moeno S. Patel M. (2020). Anti-acidogenic, anti-biofilm and slow release properties of Dodonaea viscosa var. angustifolia flavone stabilized polymeric nanoparticles. Arch. Oral Biol. 109, 104586. 10.1016/j.archoralbio.2019.104586 31630005

[B75] Serafini M. Peluso I. Raguzzini A. (2010). Flavonoids as anti-inflammatory agents. Proc. Nutr. Soc. 69 (3), 273–278. 10.1017/S002966511000162X 20569521

[B76] Seyfi P. Mostafaie A. Mansouri K. Arshadi D. Mohammadi-Motlagh H. R. Kiani A. (2010). *In-vitro* and *in-vivo* anti-angiogenesis effect of shallot (*Allium ascalonicum*): A heat-stable and flavonoid-rich fraction of shallot extract potently inhibits angiogenesis. Toxicol. *in-vitro* 24 (6), 1655–1661. 10.1016/j.tiv.2010.05.022 20570718

[B77] Shady N. H. Mostafa N. M. Fayez S. Abdel-Rahman I. M. Maher S. A. Zayed A. (2022). Mechanistic wound healing and antioxidant potential of *Moringa oleifera* seeds extract supported by metabolic profiling, in silico network design, molecular docking, and *in vivo* studies. Silico Netw. Des. Mol. Docking, Vivo Stud. Antioxidants (Basel, Switz. 11 (9), 1743. 10.3390/antiox11091743 PMC9495458 36139817

[B78] Shanthi S. Seethalakshmi S. Chamundeeswari D. Manna P. K. (2015). Evaluation of wound healing effect of *Dodonaea viscosa* Linn. by cell proliferation assay. Int. J. Pharmacogn. Phytochemical Res. 7, 559–562.

[B79] Shoulders M. D. Raines R. T. (2009). Collagen structure and stability. Annu. Rev. Biochem. 78, 929–958. 10.1146/annurev.biochem.77.032207.120833 19344236 PMC2846778

[B80] Strodtbeck F. (2001). Physiology of wound healing. Newborn Infant Nurs. Rev. 1, 43–52. 10.1053/nbin.2001.23176

[B81] Sutthammikorn N. Supajatura V. Yue H. Takahashi M. Chansakaow S. Nakano N. (2021). Topical *Gynura procumbens* as a novel therapeutic improves wound healing in diabetic mice. Plants (Basel, Switz. 10 (6), 1122. 10.3390/plants10061122 PMC8228548 34205899

[B82] Tang T. Yin L. Yang J. Shan G. (2007). Emodin, an anthraquinone derivative from Rheum officinale Baill, enhances cutaneous wound healing in rats. Eur. J. Pharmacol. 567 (3), 177–185. 10.1016/j.ejphar.2007.02.033 17540366

[B83] Tang Y. Li Y. Yu H. Gao C. Liu L. Xing M. (2014). Quercetin attenuates chronic ethanol hepatotoxicity: Implication of "free" iron uptake and release. Food Chem. Toxicol. 67, 131–138. 10.1016/j.fct.2014.02.022 24569067

[B84] Teffo L. S. Aderogba M. A. Eloff J. N. (2010). Antibacterial and antioxidant activities of four kaempferol methyl ethers isolated from Dodonaea viscosa Jacq. var. angustifolia leaf extracts. South Afr. J. Bot. 76, 25–29. 10.1016/j.sajb.2009.06.010

[B85] Upadhyay A. Chattopadhyay P. Goyary D. Mazumder P. M. Veer V. (2013). *Eleutherine indica L*. accelerates *in-vivo* cutaneous wound healing by stimulating Smad-mediated collagen production. J. Ethnopharmacol. 146 (2), 490–494. 10.1016/j.jep.2013.01.012 23337744

[B86] Van Heerden F. R. Viljoen A. M. Van Wyk B. E. (2000). The major flavonoid of *Dodonaea angustifolia* . Fitoterapia 71 (5), 602–604. 10.1016/S0367-326X(00)00201-X 11449522

[B87] Veerapur V. P. Prabhakar K. R. Thippeswamy B. S. Bansal P. Srinivasan K. K. Unnikrishnan M. K. (2010). Antidiabetic effect of Dodonaea viscosa (L). Lacq. Aerial parts in high fructose-fed insulin resistant rats: A mechanism based study. Indian J. Exp. Biol. 48 (8), 800–810. 21341538

